# A Comprehensive Transcriptional Profiling of Pepper Responses to Root-Knot Nematode

**DOI:** 10.3390/genes11121507

**Published:** 2020-12-15

**Authors:** Weiming Hu, Krista Kingsbury, Shova Mishra, Peter DiGennaro

**Affiliations:** Entomology and Nematology Department, University of Florida, Gainesville, FL 32611, USA; huw@ufl.edu (W.H.); kkingsbury@ufl.edu (K.K.); shovamishra@ufl.edu (S.M.)

**Keywords:** root-knot nematode, transcriptome, resistance, pepper

## Abstract

Genetic resistance remains a key component in integrated pest management systems. The cosmopolitan root-knot nematode (RKN; *Meloidogyne* spp.) proves a significant management challenge as virulence and pathogenicity vary among and within species. RKN greatly reduces commercial bell pepper yield, and breeding programs continuously develop cultivars to emerging nematode threats. However, there is a lack of knowledge concerning the nature and forms of nematode resistance. Defining how resistant and susceptible pepper cultivars mount defenses against RKN attacks can help inform breeding programs. Here, we characterized the transcriptional responses of the highly related resistant (Charleston Belle) and susceptible (Keystone Resistance Giant) pepper cultivars throughout early nematode infection stages. Comprehensive transcriptomic sequencing of resistant and susceptible cultivar roots with or without *Meloidogyne* *incognita* infection over three-time points; covering early penetration (1-day), through feeding site maintenance (7-days post-inoculation), produced > 300 million high quality reads. Close examination of chromosome P9, on which nematode resistance hotspots are located, showed more differentially expressed genes were upregulated in resistant cultivar at day 1 when compared to the susceptible cultivar. Our comprehensive approach to transcriptomic profiling of pepper resistance revealed novel insights into how RKN causes disease and the plant responses mounted to counter nematode attack. This work broadens the definition of resistance from a single loci concept to a more complex array of interrelated pathways. Focus on these pathways in breeding programs may provide more sustainable and enduring forms of resistance.

## 1. Introduction

Root-knot nematodes (RKN; *Meloidogyne* spp.) are capable of infecting more than 5000 plant species [1]. Among the numerous RKN species, *M. incognita*, *M. arenaria*, *M. javanica*, and *M. hapla* dominate as the agriculturally important nematode pathogens of bell pepper (*Capsicum annuum*) in the United States [2]. RKN utilize water and nutrients from the plants to complete its life cycle, resulting in decreased root and shoot growth and, ultimately, a significant reduction in pepper yield. Current RKN management practices in the field largely rely on resistant cultivars and chemical control [3]. As some dangerous chemical controls have been phased out, the challenge remains to discover effective alternative control measures, including novel forms of resistance. 

“Carolina hot” was the earliest root-knot nematode-resistant pepper line, although resistance to root-knot nematode species was unknown at the time of its release [4]. Hare (1957) defined resistance to *M. incognita* as conditioned by a single dominant gene, the *N* gene [5]. Backcrossing the *N* gene into pimiento pepper led to the subsequent release of the cultivar “Mississippi Nemaheart” which demonstrated resistance to *M. incognita* [6]. The U.S. Department of Agriculture released the first *M. incognita* resistant bell pepper, “Charleston Belle” and “Carolina Wonder”, both of which are homozygous for the *N* gene [7]. The resistance to *M. arenaria* and *M. javanica* were also conferred in “Charleston Belle” and attributed to the *N* gene, but this resistance did not extend to all critical species, including *M. hapla* [2].

Aside from the *N* gene, there are a total of nine other dominant *NBS-LRR* resistant genes to root-knot nematode in pepper cultivars, *Me1* to *Me7*, *Mech1*, and *Mech2* [8,9]. Four of the known pepper resistance genes (*Me1*, *Me3*, *Me7*, and *N*) confer resistance to a wide range of RKN and map to a 28 cM cluster on chromosome P9 [8,10]. Similar resistance gene hotspots are present in potato and tomato on chromosome P12 [11,12]. Several molecular markers associated with those resistance loci have been developed and successfully deployed in multiple breeding programs [13]. Despite extensive mapping research and molecular characterization including the mode of action, the precise location of these resistance genes remains largely unknown [14]. 

The most well-characterized resistance gene for RKN is *Mi* gene in tomato. There are two genes within the *Mi-1* loci, *Mi-1.1* and *Mi-1.2*. Only *Mi-1.2* confers resistance to root-knot nematode [15]. These genes belong to a canonical *R* gene family and share several sequences and structural motifs of the nucleotide-binding site (NBS) and leucine-rich repeat (LRR) domains [16], that are responsible for the ultimate induction of hypersensitive response (HR)-like localized cell death [17]. A few RKN effectors have been suggested as potential interacting partners [18], but the interactions of those effectors with *Mi*-mediated resistance have yet to be revealed. Importantly, *Mi-1.2* mediated resistance is not isolated to RKN but confers resistance to potato aphid [19] and whitefly [16] as well. This broader role of *Mi* in pathogen resistance and the requirement of *Rme1* upstream [20] renders elucidating nematode virulence effectors triggering *Mi-1* resistance responses difficult. This broad and indirect form of resistance disagrees with the classical “gene-for-gene” definition of resistance involving interacting pairs of Resistance and Avirulence genes [21]. This is an important point as it implies targeting a single nematode gene to develop novel resistance is limited. Indeed, reports of hypervirulent lines of nematodes able to break classical resistance abound [22,23], indicating that even successful implementation of single gene resistance is prone to quick failure under the significant genetic volatility implicit within and between RKN species [14,24,25,26,27].

Examining RKN resistance in pepper reveals a similar story to that in tomato; several loosely defined but genetically linked loci likely play broad roles in triggering resistance to the multitude of root-knot nematode species, and other pathogens’, ability to cause disease. While no single gene has yet to be characterized on the molecular level, *Me7*-mediated resistance has been suggested to include one or more *NBS-LRR* genes within an almost 400 kb region on chromosome P9 [28]. The focus of identifying resistance genes in pepper has largely been centered on the presence of canonical *NBS-LRR* structures within resistance loci and thus an inherent assumption for the requirement of the “gene-for-gene” complex governing pepper resistance. As discovered with tomato, this line of investigation can be limited in providing long term resistance in the field; other approaches to defining novel cultivar resistance are warranted. Descriptions and comprehensive analyses of transcriptional regulations upon pathogen attack have proved fertile ground for defining novel pathways and targets for sustainable resistance [29,30,31,32]. New pepper lines are continuing to be evaluated for nematode resistance, some showing significant potential [33], however, the issue remains of defining these novel forms of resistance to better understand the potential endurance and applicability of these cultivars.

Here we employed comprehensive transcriptional analyses throughout the early stages of *M. incognita* infection in susceptible and resistant interactions with bell pepper. Using closely related cultivars, over a time course of initial infection, allowed for a thorough comparison of plant responses. A focus was placed on differentially expressed genes located on chromosome P9, with the primary goal of defining the unique pepper-RKN interactions based on the plant’s ability to mount a defense. This work provides details on potential distal targets to develop resistance markers not necessarily directly involved in nematode avirulence, and keys to understanding the complexities of nematode resistance responses outside of canonical pathways.

## 2. Materials and Methods 

### 2.1. Plant and Nematode Cultures

Root-knot nematode-resistant cultivar (Res) “Charleston Belle” and susceptible cultivar (Sus) “Keystone Resistant Giant” seeds were obtained from commercial resources. Four-week-old seedlings were grown in soil and transferred into 100% sand for inoculation. *M. incognita* Race3 nematode populations were maintained on tomato plants. Eggs were extracted from tomato plant roots with 10% bleach and then centrifuged at 5000 rpm for 5 min in 40% sucrose solution to remove soil debris. Clean eggs were collected from the top layer of a 40% sucrose solution and transferred onto 2 layers of filter paper placed on a screen. The screen was placed into a dish with enough water to create a water film for hatching. In the second stage juveniles hatched from the second to fifth day were collected for inoculation.

### 2.2. Experimental Design and Sample Collection

We included resistant and susceptible cultivars with or without nematode infection in the experiment. The bell pepper cultivar “Charleston Belle” and “Keystone Resistant Giant” were the RKN resistant and susceptible cultivar respectively”. Within each cultivar, we use non-nematode infected plants as control. Each treatment was repeated as three independent biological replicates. The roots of pepper plants at day 1, 4, and 7 post-inoculation, or mock inoculation, were collected for RNA extraction for a total of 36 samples. The RNA was extracted from the roots using the plant mini-RNA extraction kit (Qiagen, Hilden, Germany) following manufacture standard procedures. The quality and quantity of the RNA were quantified using NanoDrop™ (Thermo Scientific, Wilmington, DE, USA). Illumina RNASeq libraries were prepared following standard protocols and RNA sequencing was performed using the Illumina HiSeq platform (1 × 150bp) (Illumina, San Diego, CA, USA).

### 2.3. Sequencing Processing and Gene Expression Analysis

Raw sequences were checked for quality using FastQC [34]; reads with a quality score above 30 were aligned to the pepper reference genome *Capsicum annuum* vision 1.55 [35] utilizing the program STAR (V 2.7.3a) [36]. The default mode was used except specify paired minimum overlapping read at 5. On average, 49.39% of the sequences were aligned to the reference genome for all samples. We removed one sample (CI41) which had a low mapping rate before downstream analyses (Table S1). Gene counts were summarized by HTSeq-count [37]. Differentially Expressed Genes (DEG) were calculated using the R package “DESeq2” [38]. Briefly, counts from different HiSeq runs were collapsed together using the function “collapseReplicates”, and counts less than 10 overall treatments were filtered out, and the rest of the data were normalized for DEG testing. Pairwise comparisons were made within each time point due to the variable gene counts across timepoints, the uninoculated samples were used as a control group. The function “DESeq2” estimates the size factor and dispersion parameters and then fit into a generalized linear model (negative binomial distribution). A Wald test was applied to the model with Benjamini–Hochberg (BH) adjusted *p* values; the detailed model used by DESeq2 is reported in [38]. All genes with BH adjusted *p* values < 0.05 and Log2 fold change greater than 1 were considered as DEGs in this paper. The data variation was visualized in the principal component analysis (PCA) plot, one sample from a resistant plant with nematode inoculated (CI11) were not sequenced because of low RNA quantity and quality. The number of DEG shared across time points and treatments was calculated in R, and Venn diagram plots were generated using Venny2.1 [39]. The heatmaps and PCA plots were generated on transformed count data using the function “varianceStabilizingTransformation”. Gene ontology (GO) enrichment terms were calculated to the differentially expressed genes according to the annotation “GO enrichment” tools in PlantTFDB v5.0 [40]. First, GO annotation of the DEG were collected from TAIR10 [41] and UniProtKB [42], and then significantly over-represented GO terms (*p* < 0.01) or parents of those terms were tested using topGO [43], R package version 2.42.0, and Fisher’s exact test, all genes in *Capsicum annuum* Pepper reference genome v1.55 [35] were used as the reference background. The enrichment terms of upregulated and downregulated DEG within each timepoint were separately analyzed. Transcript levels that are significantly higher or lower than those in un-inoculated resistant or susceptible cultivar samples were termed up-regulated and down-regulated, respectively. 

### 2.4. Availability of Data

The raw sequencing data and normalized abundance gene count data of all the samples in this study were deposited to NCBI with accession number GSE152857.

## 3. Results

### 3.1. Sequencing Data Quality Control

**Figure 1:**
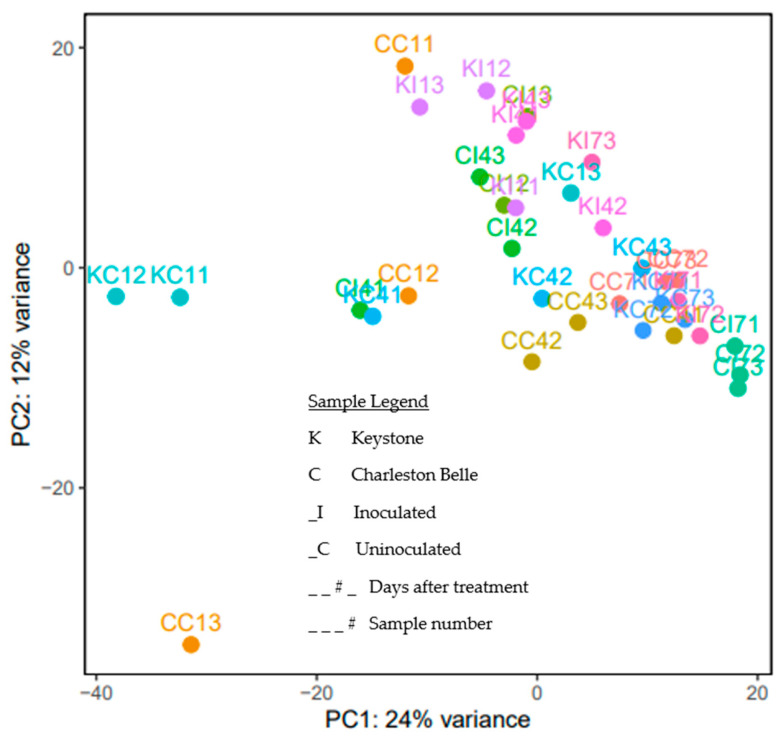
Principle component analysis of all samples by treatment shows general clustering of samples with a few outliers, Colors denote different cultivar and nematode treatments within the same timepoint.

**Figure 2:**
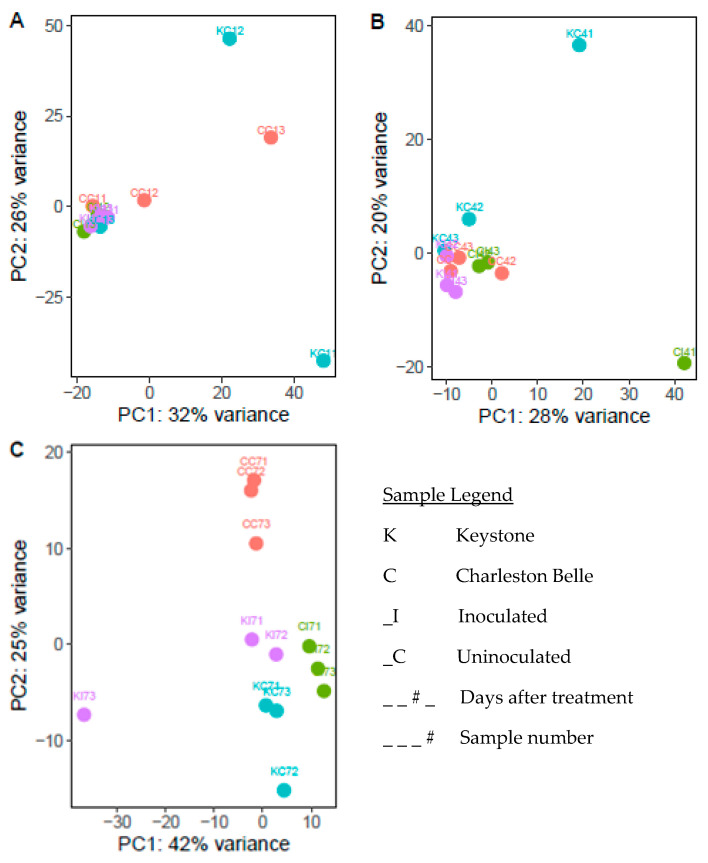
Principle component analysis of all samples within each timepoint. (**A**): day 1; (**B**): day 4; (**C**): day 7. Within each timepoint, samples with high variation were removed from downstream analyses. Colors denote different cultivar and nematode treatments within the same timepoint.

We generated a PCA plot of all samples (Figure 1), and based on the clustering, some samples with large separation from the biological replicated samples were removed in the downstream analysis. Samples not clustering based on timepoint could reflect incomplete synchronization, but we do not believe this hypothesis can fully be supported with the current amount of data. To address this issue, samples that had high variation between replicates within the same timepoint were removed (Figure 2). Specifically, KC12 and CC13 were removed from timepoint day 1 (Figure 2A); KC41 and CI41 were removed from timepoint day 4 (Figure 2B), and KI73 was removed from timepoint day7 (Figure 2C). Additionally, we did not compare gene expression between timepoints and focused on gene expression changes within each timepoint.

### 3.2. Sequencing and Gene Expression Summary

**Figure 3:**
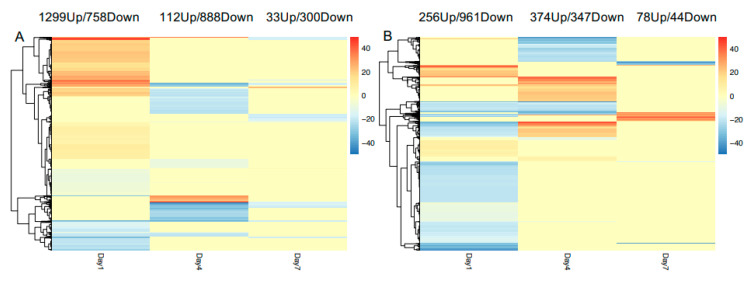
Heatmap of DEG between inoculated and uninoculated samples for each cultivar at 1, 4, and 7 days post-inoculation. (**A**): resistant cultivar; (**B**): susceptible cultivar. Up(red) and Down(blue) regulated genes are those with significantly higher and lower expression in inoculated samples, respectively. The number of the legend indicates log2 fold change.

Sequenced samples included resistant and susceptible cultivar whole roots, inoculated and uninoculated, spanning three different time points; 1, 4, and 7 days post-inoculation. With three independent biological replicates for each treatment and time point, there was a total of 36 samples. Sequencing yielded a total of 623,204,207 high-quality sequences, of which 307,810,085 mapped to the pepper reference genome, with an average mapping rate of 49.39%. The number of mapped and total sequences per sample is summarized in Table S1. There were uneven replicates for some treatments as a few samples were removed due to poor mapping rate. We also noticed that the number of reads varied between sampling timepoints, which could cause skewed results; thus, all the comparisons were made within the same timepoints that had similar read counts. We first compared inoculated to uninoculated cultivars within the same time point to characterize pepper responses to nematode infection (Figure 3). One day after inoculation showed the highest number of differentially expressed genes (DEG) in resistant and susceptible cultivars; a total of 2057 in the resistant cultivar and 1217 in susceptible. Of these 63% were upregulated in resistant plants while 21% were upregulated in susceptible plants. In contrast, at 4-days post-inoculation, the number of DEG is reduced by almost half in resistant and susceptible cultivars. Additionally, only 112 out of 1000 DEG (11%) were upregulated in resistant cultivars between inoculated and uninoculated plants. This trend continues to day 7, where 10% of only 333 DEG were upregulated in resistant plants. A similar trend of a decreasing number of DEG over time is observed in susceptible cultivar (Figure 3).

### 3.3. General Pepper Responses to Nematode Infection

**Figure 4:**
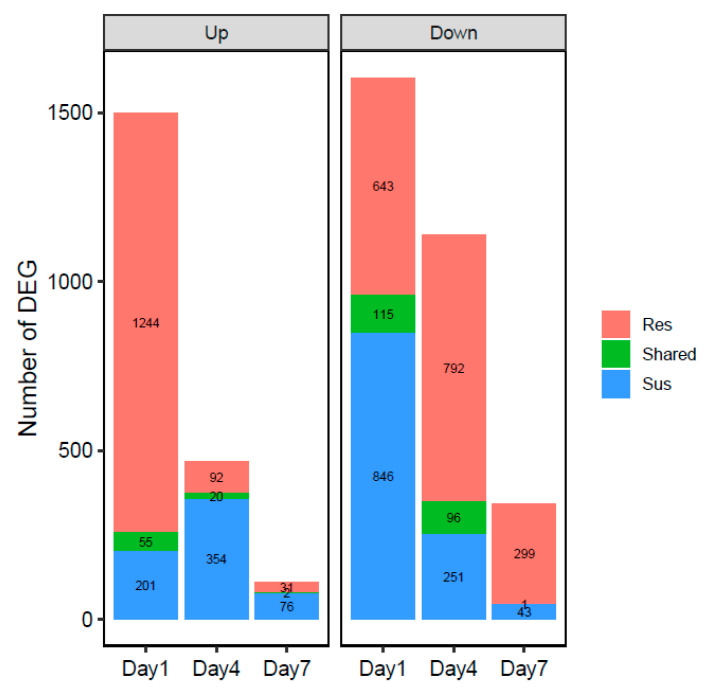
Comparison of DEG between resistant (Res) and susceptible (Sus) cultivars at 1, 4, and 7 days post-inoculation. The number in the bar graph indicated the number of DEG in each cultivar or shared (green).

To characterize general plant transcriptional responses to initial nematode infection, we compared transcriptomes of the resistant and susceptible cultivars after inoculation with *M. incognita* to uninoculated plants of the same cultivar. These genes potentially represent resistance-independent pathways and are likely general plant stress responses due to nematode infection. Comparing nematode inoculated versus uninoculated plants of the same cultivar within the same time point, and then comparing the differentially expressed genes between the cultivars (Figure 4), we identified 55 upregulated genes shared between both cultivars upon initial nematode infection. A total of 115 genes were down-regulated upon nematode infection on day 1 in both cultivars. While at day 4, there were 20 shared up-regulated genes, and 96 shared down-regulated genes (Figure 4). With less DEG identified at day 7, there were only two shared up-regulated, and one shared down-regulated genes (Figure 4). The annotation of all the shared DEG is detailed in Table S2.

### 3.4. Unique Cultivar Responses to Nematode Infection

Differentially expressed genes and their corresponding GO terms in resistant but not susceptible cultivars upon nematode infection are of specific interest as they may play important roles in defining pepper resistance versus susceptible interactions. We identified 1887 significantly and uniquely expressed genes in the resistant cultivar at 1-day post-inoculation (Figure 4), with 1244 up-regulated and 643 down-regulated. Within these genes, GO term analyses identified 231 significantly enriched terms (*p* < 0.01; Table S3), of which 163 were unique to the upregulated set of genes, and 49 were unique to the set of down-regulated genes. On day 4 and 7, fewer genes were uniquely expressed within inoculated resistant cultivars compared to inoculated susceptible cultivars, at 884 and 330 for day 4 and day 7 post-inoculation, respectively. For day 4 post-inoculation, there were a total of 33 significantly enriched GO terms for both up-regulated and down-regulated genes. Of these, only three were shared between gene sets (Table S3). On day 7 post-inoculation, there were 8 and 4 significantly enriched GO terms for up-regulated and down-regulated gene sets, respectively. There were no GO terms shared between these sets. For a full list of significantly enriched GO terms, see Table S3.

### 3.5. Temporal Variations in Cultivar Responses to Nematode Infection

**Figure 5:**
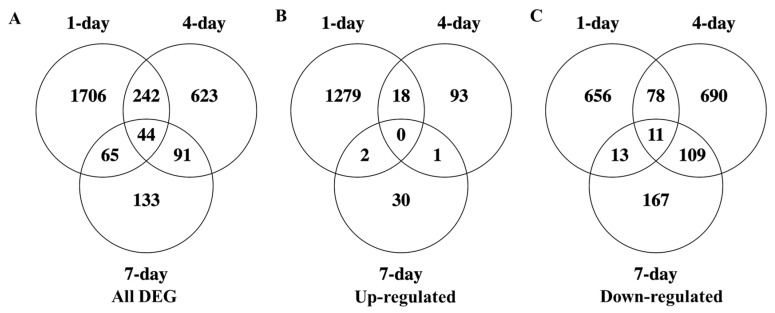
Comparison of DEG through early nematode infection in the resistant pepper cultivar. (**A**) Total number of DEG between infected and uninfected resistant cultivar at each time point. (**B**) Upregulated DEG identified between infected and uninfected resistant cultivar, (**C**) downregulated DEG identified between infected and uninfected resistant cultivar samples.

It is unclear if resistance is constantly turned on during incompatible interactions with RKN, or if it is restricted to an initial burst at the beginning stage of nematode infection. To characterize the temporal responses to nematode infection, we tracked all the DEG in resistant cultivars from day 1 to day 7, comparing inoculated with uninoculated samples. In total, there were 44 differentially expressed genes shared across all three time points between inoculated and uninoculated roots, however, 286 genes are shared between day 1 and day 4 (Figure 5A). Out of these, there were no up-regulated genes shared across all three time points, and only 18 up-regulated genes shared between day 1 and day 4 (Figure 5B). There were 11 genes down-regulated all each time point, and 89 genes were down-regulated at both day 1 and day 4 (Figure 5C). Of all the up-regulated genes shared between day 1 and day 4, one gene, on chromosome 11, is a potential NBS-coding resistance gene (Table S4). Apparent discrepancies between total DEG and combined up- and down-regulated DEG across time points are due to relative changes in DEG direction; such that while 44 genes are DEG across all time points, these genes are not consistently up- or down-regulated throughout the time course, yielding only 0 and 11 consistently up- and down-regulated genes throughout the study (Figure 5B,C).

### 3.6. Chromosome P9 and Putative RKN Resistance Loci

**Figure 6:**
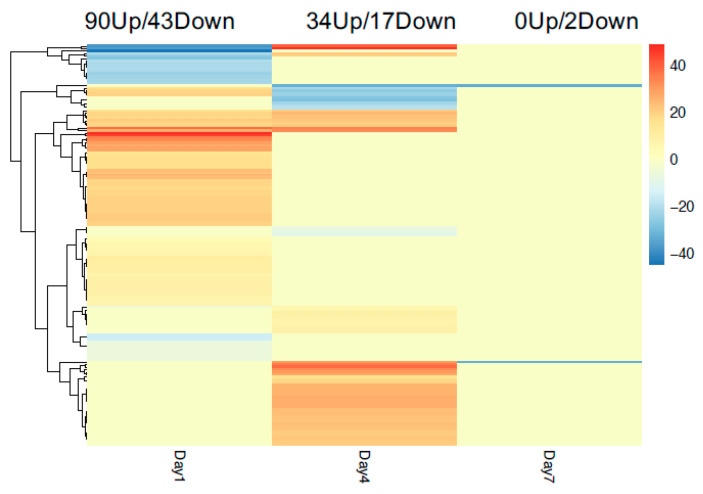
Heatmap of DEG on chromosome P9 between resistant and susceptible cultivars at 1, 4, and 7 days post-inoculation. Up(red) and Down(blue) regulated genes are those with significantly higher and lower expression in inoculated samples, respectively. The number of the legend indicates log2 fold change.

**Figure 7:**
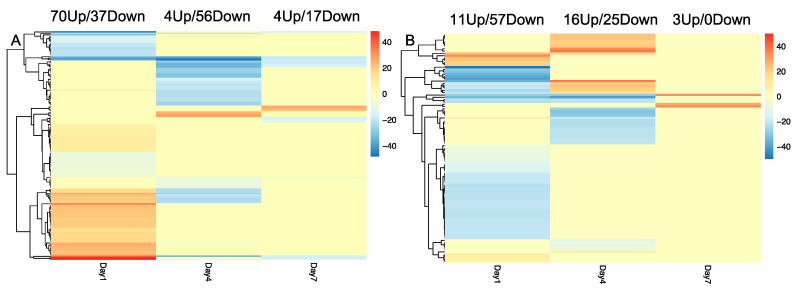
Heatmap of DEG on chromosome P9 between inoculated and uninoculated samples for each cultivar at 1, 4, and 7 days post-inoculation. (**A**): resistant cultivar; (**B**): susceptible cultivar. Up(red) and Down(blue) regulated genes are those with significantly higher and lower expression in inoculated samples, respectively. The number of the legend indicates log2 fold change.

A notable hotspot of nematode resistance loci is clustered on chromosome P9. We focused our transcriptional analysis on this chromosome and characterized the gene expression of all the genes on chromosome P9 throughout the three time points. First, we compared inoculated resistant cultivars with inoculated susceptible cultivars from the same time points. More genes were differentially expressed at day 1, and the number of DEG reduced such that at day 7, there are only 2 DEG between resistant and susceptible cultivars on chromosome P9 (Figure 6). Next, we examined DEG between inoculated and uninoculated samples within the same cultivars and time points. In the resistance cultivar, more genes were up-regulated than down-regulated on day 1 (Figure 7A). In contrast, in susceptible cultivar, there were more down-regulated genes at day 1 (Figure 7B) when comparing inoculated to uninoculated samples. Genes with opposite differential expression patterns upon nematode infection in the different cultivars may be important for resistance pathways. Assessing the DEG identified in this comparison, there were 15 genes at day 1, 6 genes at day 4, and one gene at day 7 that are differentially expressed in different directions between resistant and susceptible cultivars on chromosome P9 (Table 1). Among those genes, one gene is a putative *NBS-LRR* resistance gene, while others belong to transcription factors or kinases, or have yet to be characterized (Table 1).

A focus of many RKN resistance breeding programs is the identification of canonical *NBS-LRR* resistance genes. We mined the pepper transcriptomes of resistant and susceptible cultivars for genes that were up-regulated during infection with RKN at 1-, 4- and 7-days post-inoculation for *NBS-LRR* candidates. Based on the annotation of the pepper reference genome, the highest number of *NBS-LRR* genes were detected at day 1 (Table 2), and nearly no *NBS-LRR* genes were observed at day 7, except six *NBS-LRR* genes in resistance cultivar with nematode infection. Expectedly, the number of *NBS-LRR* DEG was less in susceptible cultivar (2) than resistance cultivar (26) at day 1 (Table 2).

## 4. Discussion

Plant pathogens elicit quantitative defense responses that can be summarized by changes in global transcriptional responses upon infection. These responses are likely to be different depending on the genetic make-up, or resistance, of the host [44]. Plant-parasitic nematodes cause a wide range of quantitative responses in plants, with some interactions resulting in the qualitative hypersensitive response which involves programmed cell death at the site of infection, as is the case with many of the natural resistance genes in tomato [14]. Largely, the molecular mechanisms of nematode resistance in pepper are poorly understood, although multiple resistance genes that may act in a qualitative response have been discovered through breeding, the exact location and mode of action of these genes are yet unknown [45]. Here we comprehensively characterized gene expression between resistant and susceptible cultivars throughout initial nematode infection to characterize quantitative resistance responses. The resistant cultivar “Charleston Belle” was derived from multiple successive backcrosses to the susceptible cultivar “Keystone Resistant Giant” after an initial cross with the progenitor resistant cultivar “Mississippi Nemaheart” [6,7]. Contrasting the transcriptomes of these two highly isogenic pepper cultivars upon initial nematode infection through early feeding site initiation allowed a detailed examination of global and canonical resistance pathways to RKN infection. In the highly studied RKN-tomato system, *Mi*-mediated resistance is induced with the first 24 h after nematode infection [16]. However, due to the ability of RKN to reproduce on resistant cultivars, albeit at significantly reduced levels [46], we sought to include transcriptional comparisons through nematode feeding site initiation at 7 days post-inoculation. Additionally, these comparisons may inform how plant responses are overcome when resistance is broken in the field [47,48,49]. 

Different resistance genes can likely function in different ways, and a majority of the known nematode resistance genes, especially the canonical *NBS-LRR R* genes, function at the early stage of the infection [14]. Genes that are upregulated upon nematode infection are often the central focus [31] when comparing gene expression data, as resistance pathways are expected to be induced. However, there can be considerable value in those genes that are down-regulated upon nematode infection, especially in a susceptible cultivar which may explain RKN success, as noted in other studies [50]. We identified 846 uniquely downregulated genes in the susceptible cultivar one day after inoculation with RKN. Of note, we identified one *NBS-LRR* gene (CA09g16930) downregulated but not differentially expressed in resistant cultivar, indicating that the pathway this gene involved in might be important for nematode infection. Another interesting gene is one Leucine-rich repeat receptor protein kinase (CA04g14230), which was upregulated in resistant cultivar but downregulated in susceptible cultivar. Similar Leucine-rich repeat serine/threonine kinase has been demonstrated to be putative receptor of an unidentified nematode-associated molecular pattern [51]. The involvement of this protein kinase in nematode resistance is not verified and worth further study. 

It is also interesting the number of differentially expressed genes is highest at the early stage of infection and then decreases as the infection progresses. There are a limited number of studies that characterize the gene expression upon nematode expression with compatible and incompatible interactions. We identified a total of 2057 differentially expressed genes in the resistant cultivar “Charleston Belle” one day after inoculation with RKN. Four days after inoculation, the number of DEG dropped to only 1000. Over 65% of the DEG on day 1 were upregulated, and this number dropped to only 11% on day 4. This result is in stark contrast to the response we observed in the susceptible cultivar where only 21% of the 1,217 DEG were upregulated at day one, and 52% of the 721 DEG were upregulated at day 4. This trend of early up-regulation of pepper genes in the resistant cultivar and down-regulation in the susceptible cultivar is accentuated when we focus on the chromosome P9, containing the known RKN resistance loci. 

Different pepper cultivars carry different arrays of the known nematode resistance genes and demonstrate very different responses to nematode infection. A cultivar carrying *Me3* has a lower juvenile penetration rate and necrosis of plant cells shortly after nematode inoculation, while a cultivar carrying *Me1* displayed an opposite response [52]. The plant defense response to RKN has been characterized as both pattern-triggered immunity and effector-triggered immunity. Pattern-recognition receptors were widely discovered to regulate nematode penetration, such as BAK1 which is a coreceptor involved in microbial perception [53], and canonical pattern-triggered immunity is also believed to be involved in nematode penetration [54]. Receptor-like kinase which has a similar pathway of microbial pattern-triggered receptors can also regulate nematode penetration [55]. These studies imply the requirement of additional genetic components outside of the known nematode resistance genes and loci. Our study examined this possibility by comparing resistant and susceptible gene expression patterns. We provide information on different sets of genes that are directly or indirectly involved in nematode infection, which is valuable for revealing the interaction mechanism of nematode infection and novel resistance discovery. 

Nematode resistance genes show similar clustered genomic structures as microbial pathogen resistance genes, such that resistance genes tend to be clustered together on one chromosome and form resistance gene hotspots [51]. In pepper, the hotspots are thought to be chromosome P9 [28]. The majority of known nematode resistance loci are localized on chromosome P9 between 248 Mb and 252 Mb [28]. There were 27 DEG within this region and most of them were upregulated when comparing inoculated and uninoculated in resistant cultivar (Table S5). However, fewer DEG (mostly downregulated) were found in the susceptible cultivar within the same genomic region (Table S5). The gene, CA09g17050 is a known *NBS-LRR* resistance gene and is upregulated in resistance cultivar, although three other *NBS-LRR* resistance genes were downregulated, indicating that not all *NBS-LRR* genes are necessarily involved in resistance responses. Another *NBS-LRR* gene, CA09g17010, was downregulated in the susceptible cultivar but upregulated in the resistant cultivar, indicating it may be a candidate resistance gene, though further validation on this gene’s activity is needed (Table S5). Unannotated genes that have opposite expression directions between resistant and susceptible cultivars may provide a novel resource for candidate resistance pathways. Other pathways not discovered, such as arginine methyltransferase, Myo-inositol oxygenase, glucuronoxylan methyltransferase might be involved in nematode penetration. The gene CA09g17960 is of great interest, as it was downregulated across all three timepoints in resistant cultivar but was upregulated 33-fold at day 7 in the susceptible cultivar. Modification of glucuronoxylan has not been reported to be directly involved in RKN. However, mutations of an enzyme involved in producing UDP-glucose resulted in small syncytia and less cyst nematode production [56]. Cyst nematode and root-knot nematode are both secondary endoparasitic nematodes, which both manipulate the plant cell abnormal growth, but use different strategies. Cyst nematode degrades the cell wall, while root-knot nematode makes cell walls thicker. Glucuronoxylan methylation genes were expressed in secondary wall-forming cells in *Arabidopsis* [57], and in resistant cultivar, this gene was downregulated ~30 times at day 1 and day 4, while not affected in susceptible cultivar until day 7. The transcriptome expression data suggested that the glucuronoxylan methylation pathway may influence a successful nematode feeding site formation.

## 5. Conclusions

Some genes are similarly up- or down-regulated in resistant and susceptible cultivars that may reveal general pathogen or nematode responses and are likely independent of specific resistance pathways. Additionally, genes that are upregulated in resistant cultivar but correspondingly downregulated in the susceptible cultivar may play important roles in regulating, or are associated with, nematode specific resistance pathways. Employing a broad approach to characterizing nematode responses in pepper allowed us to evaluate the hypothesis that susceptibility, like resistance, can also be useful to inform breeding programs. As nematode resistance can be but one of a multitude of agronomic traits used to evaluate the potential of novel pepper cultivars [58], incorporation of this data with other cultivar evaluation efforts may shed light on shared pathways and loci responsible and sufficient for an array of biotic stress responses. As nematode resistance loci are not always restricted to a single pathogen pressure response [59], we must place resistance responses in broader contexts.

## Figures and Tables

**Table 1:** Select directionally DEG located within chromosome P9.

GeneID	Resistant ^1^	Susceptible ^1^	Time Point	Function
CA09g00350	19.97	−20	day 1	Rac GTPase activating protein 1
CA09g07060	−13.44	23.41	day 1	Unknown protein
CA09g08070	17	−22.02	day 1	subtilisin-like protease-like
CA09g08670	37.37	−31.48	day 1	Acetyl-CoA carboxylase, putative
CA09g08790	20.61	−20.69	day 1	Protein binding protein, putative
CA09g09700	20.99	−21.9	day 1	Multicopper oxidase, putative
CA09g09910	−16.65	19.09	day 1	Tetratricopeptide repeat-containing protein
CA09g11690	21.56	−19.29	day 1	STY-L protein
CA09g11780	21.24	−20.79	day 1	3-ketoacyl-CoA reductase 1
CA09g14450	20.12	−20.88	day 1	WD repeat-containing protein 26-like isoform X1
CA09g15640	16.25	−13.7	day 1	ATP synthase subunit α
CA09g16660	15.2	−20.24	day 1	Unknown protein
CA09g16770	7.72	−9.87	day 1	Unknown protein
CA09g16890	20.97	−19.79	day 1	Unknown protein
CA09g17010	48.07	−39.53	day 1	BED finger-nbs-lrr resistance protein
CA09g04320	28.3	−27.12	day 4	F-box/kelch-repeat protein At3g23880-like
CA09g05170	−21.52	19.29	day 4	GRAS family transcription factor
CA09g12300	−36.36	33.82	day 4	Purple acid phosphatase
CA09g15320	−33.87	37.99	day 4	Putative serine/threonine protein kinase
CA09g16890	−21.29	21.94	day 4	Unknown protein
CA09g18730	−21.59	22.2	day 4	Senescence-associated protein
CA09g17960	−18.13	33	day 7	glucuronoxylan 4-O-methyltransferase 2-like

^1^ Resistant and Susceptible values are expression fold changes between infected and uninfected samples.

**Table 2:** The regulation of potential resistance DEG across time points.

Cultivar and Resistance Type	Time and Number of Genes		
Resistant	day 1	day 4	day 7
*NBS-LRR* resistance genes	26	9	6
Nematode resistance genes	4	3	0
Susceptible	day 1	day 4	day 7
*NBS-LRR* resistance genes	2	1	0
Nematode resistance genes	7	7	0

## Supplementary Materials

The following are available online at https://www.mdpi.com/2073-4425/11/12/1507/s1, Table S1. Summary of the sequence reads and mapping rate; Table S2. DEG shared between resistant and susceptible cultivars; Table S3. Significantly enriched GO terms between DEG of inoculated resistant and susceptible cultivars. Shared up- and downregulated genes across time points in resistance cultivar; Table S4. Shared up- and down-regulated genes across time points in resistance cultivar. Table S5. DEG within known resistance loci on chromosome P9.
