# Supplementary material for: A Comprehensive Transcriptional Profiling of Pepper Responses to Root-Knot Nematode
Source: Genes (Basel). 2020 Dec 15;11(12):1507. doi: 10.3390/genes11121507 (PMC7765216; doi:10.3390/genes11121507)
Supplement: Supplementary file 1 [file genes-11-01507-s001.zip › Table S1.docx]

Table S1 Summary of the sequence reads and mapping rate

| Sample | Total reads | Mapped reads | Mapping rate |
| --- | --- | --- | --- |
| CC11 | 24052339 | 8401164 | 34.92868 |
| CC12 | 23569888 | 14600777 | 61.94674 |
| CC13 | 23757839 | 14781413 | 62.21699 |
| CC41 | 23760725 | 14577049 | 61.34934 |
| CC42 | 23624145 | 14902126 | 63.08006 |
| CC43 | 23826507 | 14050154 | 58.96858 |
| CC71 | 22993811 | 10491893 | 45.6292 |
| CC72 | 24072169 | 12018913 | 49.92867 |
| CC73 | 23899728 | 15694856 | 65.6696 |
| CI12 | 12531760 | 3685627 | 29.41029 |
| CI13 | 13610190 | 3999164 | 29.3836 |
| CI41 | 13855920 | 482511 | 3.482345 |
| CI42 | 12326772 | 8893305 | 72.14626 |
| CI43 | 16274318 | 9641747 | 59.24517 |
| CI71 | 14223166 | 9438991 | 66.3635 |
| CI72 | 14418093 | 11108443 | 77.04516 |
| CI73 | 13670173 | 10026656 | 73.34696 |
| KC11 | 647806 | 70654 | 10.90666 |
| KC12 | 2472920 | 273449 | 11.05774 |
| KC13 | 9800336 | 2913764 | 29.73127 |
| KC41 | 7201734 | 4397267 | 61.05845 |
| KC42 | 9095266 | 5687740 | 62.53517 |
| KC43 | 12833588 | 7726885 | 60.2083 |
| KC71 | 9914935 | 6168455 | 62.21377 |
| KC72 | 9157356 | 7184768 | 78.45898 |
| KC73 | 9832536 | 8076765 | 82.14325 |
| KI11 | 11609111 | 5456075 | 46.99822 |
| KI12 | 54585509 | 13278634 | 24.3263 |
| KI13 | 45316183 | 16353353 | 36.08723 |
| KI41 | 887046 | 331491 | 37.37022 |
| KI42 | 4417015 | 1758717 | 39.81687 |
| KI43 | 29980232 | 11888534 | 39.65458 |
| KI71 | 13035655 | 8551303 | 65.59934 |
| KI72 | 10500991 | 7790789 | 74.19099 |
| KI73 | 57448445 | 23106653 | 40.22155 |
